# Paths of thoracic epidural catheters in children undergoing the Nuss procedure for pectus excavatum repair

**DOI:** 10.1007/s00540-022-03048-5

**Published:** 2022-03-04

**Authors:** Kanna Nakamura, Ayanori Sugita, Shuichi Sekiya, Akira Kitamura, Hiromasa Mitsuhata, Keisuke Yamaguchi, Masakazu Hayashida

**Affiliations:** 1 Department of Anesthesiology, Juntendo Tokyo Koto Geriatric Medical Center, 3-3-20 Shinsuna, Kohtoh-ku, Tokyo, 136-0075 Japan; 2 Department of Reconstructive Surgery, Tokyo Metropolitan Police Hospital, 4-22-1 Nakano, Tokyo, Nakano-ku 164-8521 Japan; 3 Department of Anesthesiology, Saitama Medical University International Medical Center, 1397-1 Yamane, Hidaka-Shi, Saitama 350-1298 Japan; 4 Department of Anesthesiology and Pain Medicine, Juntendo University School of Medicine, 2-1-1 Hongo, Bunkyo-ku, Tokyo 113-8421 Japan

**Keywords:** Catheter, Child, Epidural anesthesia, Epidural space, Pectus excavatum

## Abstract

**Purpose:**

To investigate the paths of thoracic epidural catheters in children, this retrospective study was performed.

**Methods:**

We investigated 73 children aged 4 to 12 (mean ± SD 7.8 ± 2.3) years, who underwent the Nuss procedure for pectus excavatum repair under combined general and epidural anesthesia over a 5-year period at Tokyo Metropolitan Police Hospital. Following induction of general anesthesia, we inserted a radiopaque epidural catheter via the T5/6 or T6/7 interspace and advanced for 5 cm cephalad in the thoracic epidural space. We evaluated the paths of the epidural catheters on plain chest radiographs after surgery.

**Results:**

The median level for the catheter tip location was T3 (range C6–T7), while the median number of vertebrae crossed by the catheter tips was 2.5. In most children, the catheters advanced straight for the first 2–3 cm (1–1.5 vertebrae) in the thoracic epidural space. However, they continued to advance straight in only 25 children, while they exhibited curved or coiled paths in the remaining 48. The catheter tips were located at higher levels in children with straight epidural catheter paths [median (range) T2 (C6–T4)] than in those with curved or coiled paths after the initial 2–3 cm [median (range) T4 (T2–T7)] (*p* < 0.0001).

**Conclusions:**

Our findings indicate that the course of epidural catheters in children is unpredictable after the first 2–3 cm in the thoracic epidural space. Clinicians should be aware of such findings, although further studies are required for confirmation.

## Introduction

The Nuss procedure for the repair of pectus excavatum in children involves thoracoscopy-guided placement of one to three steel bars behind the sternum and ribs through small incisions placed on both sides of the chest [1]. Although this procedure is categorized as a minimally invasive procedure, postoperative pain is significant because of forceful distortion of bony structures [1]. In some institutions, epidural analgesia is used to control postoperative pain [1, 2]. A previous study reported that epidural analgesia is superior to patient-controlled analgesia with opioids in terms of efficacy and the quality of pain control [2]. For successful epidural catheterization in children, adequate knowledge regarding the anatomy of the posterior compartment of the epidural space is essential.

At the lumbar and lower thoracic levels in adults, posterior epidural fat is discontinuous between adjacent vertebral segments and is separated by areas of contact between the dura and bony lamina [3–5]. Consequently, lumbar epidural catheters are less likely to advance in a straight line [4, 6]. On the other hand, at the mid-thoracic and upper thoracic levels, a thin layer of continuous fat is present between the dura and lamina and often extends from one segment to the other [3–5]. This incomplete segmentation at the mid-thoracic and upper thoracic levels facilitates straight advancement of thoracic epidural catheters. [4, 6]. At 2 years of age, a continuous layer of abundant posterior epidural fat extends from one segment to the other even at lumbosacral levels, with the adult pattern of segmentation of the lumbar posterior epidural space becoming evident by 10 years of age [3]. Therefore, straight passage of epidural catheters can be facilitated even at lumbosacral levels in young children [7].

At our institution, we use postoperative epidural analgesia for pain control in patients who undergo the Nuss procedure. Epidural catheters are generally inserted via the T5/6 or T6/7 interspace, with the aim of positioning the catheter tip at the T2 level or below for adequate anesthesia during thoracic surgery [8]. We usually advance thoracic epidural catheters for 5 cm into the epidural space, considering that advancement for < 4 cm increases the risk of catheter dislodgement [9]. The paths of both lumbar and thoracic epidural catheters have been subjected to detailed radiographic investigations in adult patients [6]. However, to our knowledge, the paths of blindly inserted thoracic epidural catheters have not been investigated in children, although studies have assessed the paths of thoracic epidural catheters inserted indirectly via the caudal or lumbar route [10, 11] or under guidance from ultrasonography or nerve stimulation [12, 13].

Accordingly, the aim of the present retrospective study was to investigate the paths of thoracic epidural catheters inserted up to 5 cm in the epidural space using standard blind methods in children. The study hypothesis was that epidural catheters can be easily advanced in a straight line in the thoracic epidural space of children because of incomplete segmentation of the posterior epidural space.

## Materials and methods

This retrospective study was approved by the Institutional Review Board of Tokyo Metropolitan Police Hospital (approval number 16-1), which waived the need for written informed consent.

We retrospectively reviewed data from 90 children who underwent the Nuss procedure for pectus excavatum repair under combined general and epidural anesthesia between 2006 and 2010 at Tokyo Metropolitan Police Hospital. We finally investigated 73 children (47 boys and 26 girls) aged 4 to 12 (mean ± SD 7.8 ± 2.3) years in whom radiopaque epidural catheters were inserted via the T5/6 or T6/7 interspace, after excluding 17 children in whom radiolucent epidural catheters were used or epidural catheters were inserted via an interspace other than the T5/6 or T6/7 interspace.

Following the induction of general anesthesia, the patient was placed in the lateral decubitus position. Epidural catheterization was performed by one of three experienced anesthesiologists. The T5/6 or T6/7 interspace was identified by using a line joining the inferior angles of the scapulae as a landmark for T7 [9]. A 5-cm, 18G epidural needle (Arrow International, Reading, Pennsylvania) was inserted using conventional blind methods. Once the epidural space was reached, a radiopaque 20G catheter (Flex tip plus™; Arrow International) was threaded until a sufficient length had entered the space. The needle was withdrawn, following which the catheter was withdrawn such that a length of 5 cm remained in the epidural space regardless of the age and body height. The catheter tip position was aimed at the T2 level or below by inserting epidural catheters via the T6/7 interspace in children 110 cm in height or shorter, and via the T5/6 in children taller than 110 cm, based on our own experience. The catheter was secured with a transparent adhesive drape and an elastic adhesive tape.

Immediately after completion of the Nuss procedure, plain anteroposterior and lateral chest radiographs were acquired to confirm the absence of respiratory complications and evaluate the path of the epidural catheter. Six parameters were recorded as follows: (1) actual catheter insertion point on the skin, (2) actual epidural space insertion point, (3) direction of catheter advancement immediately after entry in the epidural space, (4) position and direction of the catheter tip, (5) pattern of the catheter path, and (6) distance traveled by the catheter tip in the epidural space.

All data were statistically analyzed using analysis of variance, the Wilcoxon signed-rank test, the Mann–Whitney *U* test, the Kruskal–Wallis test, linear regression analysis, and Fisher’s exact test, as appropriate. A *p* value of < 0.05 was considered statistically significant.

## Results

Demographic and catheterization data are shown in Table 1. Linear regression analyses revealed that the skin-to-epidural space distance (SED; cm) correlated with the patient’s age (*r* = 0.48, *p* < 0.0001), body height (BH; cm; *r* = 0.51, *p* < 0.0001), and body weight (BW; kg; *r* = 0.48, *p* < 0.0001). The equations were as follows: SED = 0.11 × Age (years) + 2.4, SED = 0.019 × BH (cm) + 0.8, and SED = 0.033 × BW (kg) + 2.4.

**Table 1:** Demographic and catheterization data for children who underwent the Nuss procedure for pectus excavatum repair under combined general and epidural anesthesia

Sex, M:F	47:26
Age (year), mean ± SD (range)	7.8 ± 2.3 (4–12)
Body height (cm), mean ± SD (range)	128 ± 13 (102–159)
Body weight (kg), mean ± SD, (range)	24.5 ± 7.2 (16–47)
Intended insertion points	T5/6 (*n* = 62), T6/7 (*n* = 11)
Median [quartiles] (range)	T5/6 [T5/6, T5/6] (T5/6–T6/7)
Actual skin insertion points	T3/4 (*n* = 3), T4/5 (*n* = 19), T5/6 (*n* = 38), T6/7 (*n* = 13)
Median [quartiles] (range)	T5/6 [T4/5, T5/6] (T3/4–T6/7)
Actual epidural space insertion points	T3/4 (*n* = 5), T4/5 (*n* = 20), T5/6 (*n* = 35), T6/7 (*n* = 13)
Median [quartiles] (range)	T5/6 [T4/5, T5/6] (T3/4–T6/7)
Catheter direction immediately after entry in the epidural space	Cephalad (*n* = 72), Caudad (*n* = 1)
Catheter tip direction	Cephalad (*n* = 67), Caudad (*n* = 6)
Catheter tip position (vertebral level)	C6 (*n* = 1), C7 (*n* = 4), T1 (*n* = 7), T2 (*n* = 12), T3 (*n* = 23), T4 (*n* = 18), T5 (*n* = 7), T7 (*n* = 1)
Median [quartiles] (range)	T3 [T2, T4] (C6–T7)
Number of vertebrae crossed by catheter tips	5 (*n* = 1), 4.5 (*n* = 5), 4 (*n* = 15), 3.5 (*n* = 1), 3 (*n* = 13), 2.5 (*n* = 10), 2 (*n* = 14), 1.5 (*n* = 10), 1 (*n* = 2), 0.5 (*n* = 1), − 0.5 (*n* = 1)
Median [quartiles] (range)	+ 2.5 [+ 2, + 4] (− 0.5 to + 5)

Data are expressed as number, mean ± standard deviation (range), or median [quartiles] (range)

The intended insertion point was the T5/6 (*n* = 62) or T6/7 (*n* = 11) interspace. The epidural space was reached via a median (*n* = 67) or paramedian approach (*n* = 6), using the loss-of-resistance method (*n* = 53) or the hanging drop method (*n* = 20) (Table 1). The actual, radiographically confirmed catheter insertion points on the skin were in concordance with the intended insertion points in 47 children. In the remaining children, the insertion points were two segments higher (*n* = 3), one segment higher (*n* = 20), or one segment lower (*n* = 3) than the intended point. Similarly, the actual epidural space insertion points were in concordance with the intended insertion points in 44 children, whereas they were two segments higher (*n* = 5), one segment higher (*n* = 21), and one segment lower (*n* = 3) in the remaining children. Overall, the actual skin and epidural space insertion points were higher than the intended insertion point (*p* = 0.0004 and *p* < 0.0001, respectively; Wilcoxon test). The median level for the catheter tip location was T3 (range C6–T7), while the median number of vertebrae crossed by the tips was 2.5. The catheter tips were directed cephalad and caudad in 67 and six children, respectively (Table 1).

**Fig. 1:**
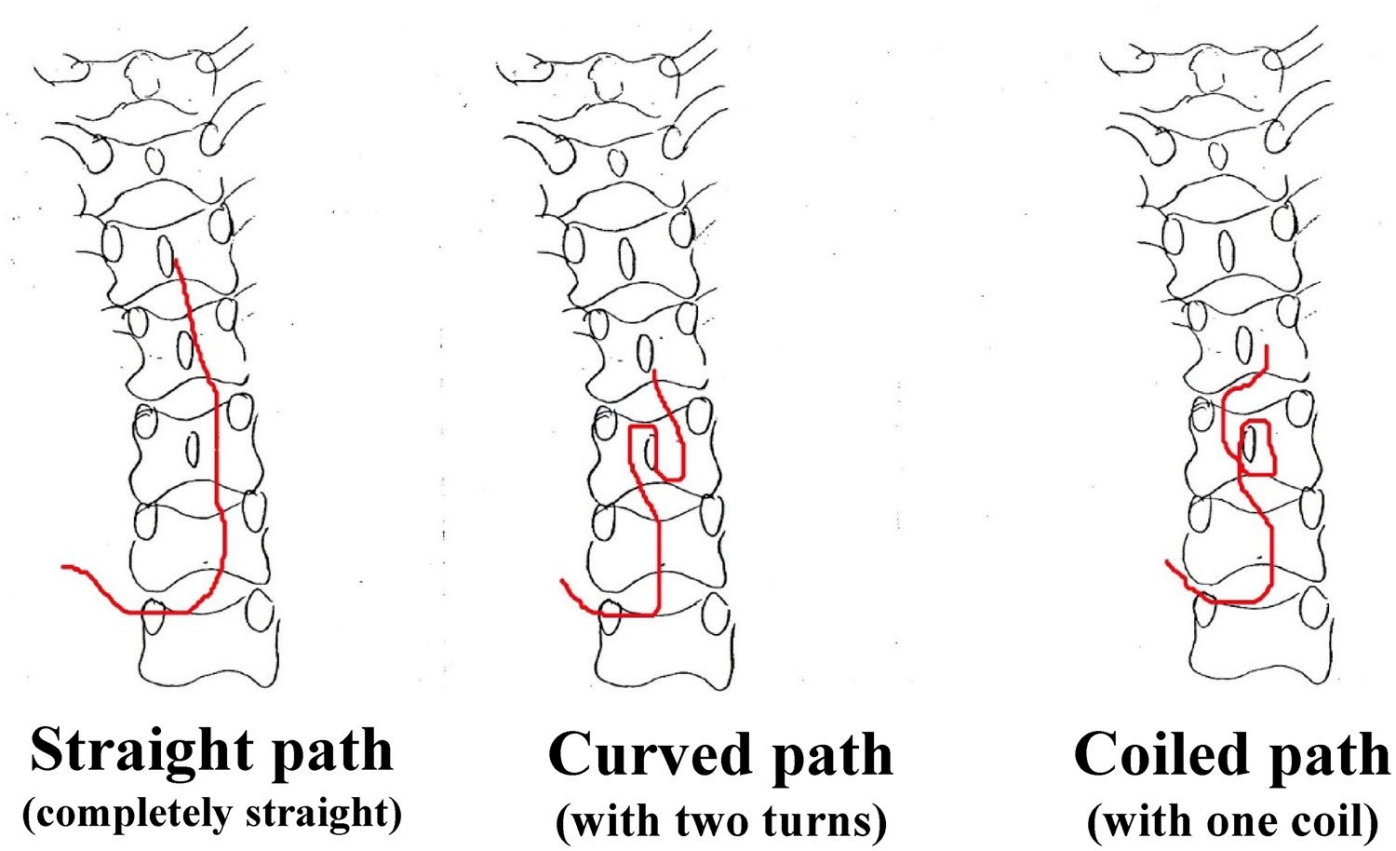
Paths of thoracic epidural catheters in children. The catheter paths can be divided into three patterns: straight, curved, and coiled. When the axis of the path exhibited a deviation of > 90° from the vertebral midline, the path was classified as a coiled or curved path. When the path axis did not show deviation of > 90°, the path was classified as a straight path (completely straight: < 30° deviation, almost straight: 30°–90° deviation). The left, middle, and right panels show examples of a completely straight path, a curved path with two turns, and a coiled path with one coil, respectively

Immediately after entry in the epidural space, the catheters advanced in a cephalad direction in 72 children and a caudad direction in one child (Table 1). Although the catheters advanced straight for the first 2–3 cm (1–1.5 vertebrae) in the epidural space of most (71/73) children, they continued to advance straight only in a third (25/73) of the children. In the remaining two thirds (48/73), they advanced in curved or coiled paths after the first 2–3 cm (Fig. 1, Table 2). According to the observed course, the catheter path were divided into three patterns: straight (*n* = 25), curved (*n* = 19), and coiled (*n* = 29; Fig. 1, Table 2). When the axis of the path exhibited a deviation of > 90° from the vertebral midline, the path was classified as a coiled or curved path. When the path axis did not show deviation of > 90°, the path was classified as a straight path (completely straight: < 30° deviation, almost straight: 30°–90° deviation). There were no significant differences in age, BH, or BW among children with straight, curved, and coiled paths (*p* = 0.78, *p* = 0.99, and *p* = 0.84, respectively; ANOVA; Table 2). Furthermore, the three groups showed no significant differences in the intended insertion point, actual skin insertion point, and actual epidural space insertion point (*p* = 0.64, *p* = 0.37, and *p* = 0.26, respectively; Kruskal–Wallis test). However, there were significant differences in the position of the catheter tip and the number of vertebrae crossed by the catheter tip (*p* < 0.0001 and *p* < 0.0001, respectively; Kruskal–Wallis test).

**Table 2:** Trichotomized or dichotomized classifications for the paths of thoracic epidural catheters in children

Catheter paths	Detailed catheter paths	Age (years)	Height (cm)	Weight (kg)	Intended insertion point	Actual skin insertion point	Actual epidural space insertion point	Catheter tip position (vertebral level)	Number of vertebrae crossed by catheter tips
Straight (*n* = 25)	Completely straight (*n* = 15), Almost straight (*n* = 10)	7.6 ± 2.4 (4–11)	128 ± 14 (109–151)	24.4 ± 7.4 (16–47)	T5/6 [T5/6, T5/6] (T5/6–T6/7)	T5/6 [T4/5, T5/6] (T3/4–T6/7)	T5/6 [T4/5, T5/6] (T3/4–T6/7)	T2 [T1, T2] (C6–T4)	+ 4 [+ 4, + 4] (+ 2 to + 5)
Curved (*n* = 19)	One turn (*n* = 3) Two turns (*n* = 14) Three turns (*n* = 2)	7.9 ± 2.5 (5–12)	128 ± 12 (111–154)	23.8 ± 5.3 (16–37)	T5/6 [T5/6, T5/6] (T5/6–T6/7)	T5/6 [T4/5, T5/6] (T4/5–T6/7)	T4/5 [T4/5, T5/6] (T3/4–T6/7)	T4 [T3, T4] (T3–T7)	+ 2 [+ 1.75, + 2.5] (− 0.5 to + 3)
Coiled (*n* = 29)	One coil (*n* = 10) Two coils (*n* = 12) Three coils (*n* = 7)	8.0 ± 2.2 (4–12)	128 ± 14 (102–159)	25.1 ± 8.3 (16–47)	T5/6 [T5/6, T5/6] (T5/6–T6/7)	T5/6 [T5/6, T6/7] (T3/4–T6/7)	T5/6 [T4/5, T6/7] (T3/4–T6/7)	T4 [T3, T4] (T2–T5)	+ 2.5 [+ 1.5, + 3] (+ 0.5 to + 4)
Curved or Coiled (*n* = 48)	Curved (*n* = 19) Coiled (*n* = 29)	8.0 ± 2.3 (4–12)	128 ± 13 (102–159)	24.6 ± 7.2 (16–47)	T5/6 [T5/6, T5/6] (T5/6–T6/7)	T5/6 [T4/5, T5/6] (T3/4–T6/7)	T5/6 [T4/5, T5/6] (T3/4–T6/7)	T4 [T3, T4] (T2–T7)	+ 2 [+ 1.5, + 2.75] (− 0.5 to + 4)

Data are expressed as mean ± standard deviation (range) or median [quartiles] (range)

When the axis of the path exhibited a deviation of > 90° from the vertebral midline, the path was classified as a coiled or curved path. When the path axis did not show deviation of > 90°, the path was classified as a straight path (completely straight: < 30° deviation, almost straight: 30°–90° deviation)

Considering the lack of significant differences in the intended insertion point, actual skin insertion point, actual epidural space insertion point, catheter tip position, and number of vertebrae crossed by the catheter tip between children with curved paths and those with coiled paths (*p* = 0.78, *p* = 0.23, *p* = 0.13, *p* = 0.93, and *p* = 0.32, respectively; Mann–Whitney test), the children were dichotomized into a straight group (*n* = 25) and a curved/coiled group (*n* = 48) for subsequent statistical analyses (Table 2). The two groups showed no significant differences in the intended insertion point, actual skin insertion point, and actual epidural space insertion point (*p* = 0.45, *p* = 0.50, and *p* = 0.71, respectively; Mann–Whitney test). However, the median number of vertebrae crossed by the catheter tip was significantly more in the straight group than in the curved/coiled group (+ 4 vs. + 2, *p* < 0.0001; Mann–Whitney test). Moreover, the catheter tips were located at higher levels in children with straight paths [median (range) T2 (C6–T4)] than in those with curved or coiled paths [median (range) T4 (T2–T7); *p* < 0.0001, Mann–Whitney test]. The frequency of placement at the T1 level or above was significantly higher in the straight group than in the curved/coiled group (12/25 vs. 0/48, *p* < 0.0001; Fisher’s exact test). Further, the frequency of placement at the C6 or C7 level was significantly higher in the straight group (5/25 vs. 0/48, *p* = 0.0035; Fisher’s exact test). The positioning of the catheter tips at the C6 or C7 level (*n* = 5) in the straight group was associated with actual skin puncture points more cephalad than the intended points (T3/4 [*n* = 1] or T4/5 [*n* = 3] instead of intended T5/6) or a relatively short BH and thin body (BH, 110.6 cm; BW, 15.7 kg corresponding to − 16.6% of the standard BW [14]; actual puncture via T5/6 as intended [*n* = 1]).

The epidural catheter was directed toward the intervertebral foramen in two children. Horner’s syndrome, which was not associated with the catheter tip position, was recognized in 13 children immediately after surgery. However, there were no serious neurological complications related to epidural anesthesia.

## Discussion

In the present study, we found that the course of epidural catheters in children who underwent the Nuss procedure under combined general and epidural anesthesia was unpredictable after the first 2–3 cm in the thoracic epidural space. Moreover, the skin-to-epidural space distance was significantly correlated with the age, height, and weight of the children, similar to the findings in a previous study [15]. In one third (26/73) of children, the actual epidural space insertion points were located more cephalad than the intended insertion points when the line joining the inferior angles of the scapulae was used as a landmark for T7 [9]. This highlights the unreliability of such moveable landmarks in children placed in the lateral position under general anesthesia. Ideally, this landmark should have been used to mark T7 in the sitting or standing position before anesthesia. Alternatively, insertion points should have been determined on the basis of fixed bony landmarks such as the C7 prominence.

Before the study, we hypothesized that thoracic epidural catheters would move in a straight line in children because of incomplete segmentation of the posterior compartment of the thoracic epidural space [3, 4]. However, curving or coiling of catheters after the first 2–3 cm in the epidural space was observed in two thirds (48/73) of children. These findings suggest an unpredictable course after the initial 2–3 cm. We used Arrow reinforced catheters in the present study. This catheter contains a stainless-steel coil impregnated in soft polyurethane; thus, it can easily change direction when it encounters an obstacle [16]. Consequently, the incidence of complications, including paresthesia and venipuncture, is significantly lower with this catheter than with other stiffer catheters [17, 18]. However, the flexibility of this catheter could have partially contributed to the high incidence of curving or coiling observed in our study. In addition, the considerably narrow epidural space in children relative to that in adults [3] may have interfered with straight catheter advancement.

In the present study, epidural catheters were advanced for 5 cm in the thoracic epidural space. In most (71/73) of children, the catheters advanced straight for the first 2–3 cm (1–1.5 vertebrae) in the epidural space; this implies that even flexible catheters usually advance straight for an initial small distance after entering the epidural space. After the first 2–3 cm, however, the catheters continued to advance straight in only a third (25/73) of children. Although this was a desirable path, very high placement of the catheter tips (T1 level or above, or even C6 or C7 level) was more frequent when the catheters moved in a straight course than when they moved in curved or coiled courses. The very high placement did not occur when the catheters curved or coiled after straight advancement for 2–3 cm. Therefore, one may argue that catheter advancement for 2–3 cm, not 5 cm, is adequate because it allows a straight path and helps to avoid very high placement. However, the risk of the catheter dislodgement is increased when catheters are advanced only for < 4 cm in the epidural space [9]. Further, whilst we aimed catheter positioning at the T2 level or below, this aim was mostly achieved by advancing catheters for 5 cm (median tip positions; T3 in the total cohort, T2 in the straight group, and T4 in the curved/coiled group). Therefore, catheter advancement for 4–5 cm in the epidural space seemed acceptable also in children, although clinicians should be aware of the possibility of too high placement of the catheter tip that can occur in some children, especially when the actual skin puncture points more cephalad than the intended points are erroneously determined based on unreliable landmarks and/or a child has a relatively short height (around 110 cm) and a thin body.

This study only investigated the paths of flexible radiopaque epidural catheters. Consequently, it remains unclear whether stiffer radiolucent catheters advance straight or curved/coiled in the thoracic epidural space in children. Further studies using a radiocontrast agent should address this limitation.

In conclusion, our findings indicate that the course of thoracic epidural catheters in children is unpredictable after the first 2–3 cm in the thoracic epidural space. Clinicians should be aware of such findings, although further studies are required for confirmation.
